# Urinary presepsin can efficiently detect T-cell-mediated rejection in patients who have undergone kidney transplantation

**DOI:** 10.1007/s10157-025-02672-1

**Published:** 2025-04-05

**Authors:** Tomohiro Kawazoe, Akihito Tanaka, Kazuhiro Furuhashi, Keita Hattori, Chikao Onogi, Akiko Owaki, Akihisa Kato, Yu Watanabe, Eri Koshi-Ito, Noritoshi Kato, Tomoki Kosugi, Yuta Sano, Shohei Ishida, Shoichi Maruyama

**Affiliations:** 1 Department of Nephrology, Nagoya University Graduate School of Medicine, 65 Tsurumai-Cho, Showa-Ku, Nagoya-City, Aichi 466-8550 Japan; 2 Department of Nephrology, Nagoya University Hospital, 65 Tsurumai-Cho, Showa-Ku, Nagoya-City, Aichi, 466-8550 Japan; 3 Department of Urology, Nagoya University Hospital, 65 Tsurumai-Cho, Showa-Ku, Nagoya-City, Aichi 466-8550 Japan

**Keywords:** Kidney transplantation, Rejection, T-cell-mediated rejection, Urinary presepsin

## Abstract

Urinary presepsin (uPSEP) is a marker of tubular interstitial injury. For patients who have undergone kidney transplantation, the early diagnosis of rejection is important to early treatment and preservation of the transplanted kidney function. We investigated whether uPSEP is useful for predicting T-cell-mediated rejection (TCMR). Patients who underwent graft biopsy in 2020 and 2023 after kidney transplantation at our hospital were included. We excluded protocol biopsy samples obtained at 1 h. We measured uPSEP and divided the patients into groups based on the presence or absence of TCMR; then, group comparisons were performed. A total of 39 patients (17 female and 22 male patients) with a median age of 57 years (interquartile range [IQR], 46.5–63 years) at the time of biopsy were included. Thirty-one patients underwent protocol biopsies and eight underwent episode biopsies. TCMR occurred in three patients. The uPSEP value of the TCMR group was 6788.63 ng/gCr (IQR, 5374.57–9931.87 ng/gCr), and that of the non-TCMR group was 777.61 ng/gCr (IQR, 321.57–1299.63 ng/gCr) (*P* < 0.01). The receiver-operating characteristic curve for predicting TCMR had a cutoff value of 3961 ng/gCr and an area under the curve of 0.982 (95% confidence interval [CI], 0.942–1). The odds ratio of TCMR based on uPSEP (per 1000-ng/gCr increase in uPSEP) was 1.90 (95% CI, 1.10–3.28; *P* = 0.02). uPSEP levels may predict TCMR with high accuracy.

## Introduction

A kidney biopsy is the gold standard for diagnosing kidney disease because it provides important information; however, it is associated with risks such as bleeding complications [1, 2]. Additionally, kidney biopsy results are not quickly obtained; therefore, when a quick diagnosis and treatment decision are necessary, a kidney biopsy alone may not be sufficient. Therefore, novel, highly accurate, minimally invasive, and immediately available biomarkers that can replace or complement the kidney biopsy are urgently needed [3–5]. We focused on urinary presepsin (uPSEP). Blood presepsin (PSEP) measurements are covered by health insurance in Japan and are considered useful for the early diagnosis of sepsis. In this context, we discovered that uPSEP is an extremely good biomarker that can detect interstitial inflammation of the kidney [6].

A graft biopsy provides important information regarding patients who have undergone kidney transplantation [7, 8]. For example, it can confirm the recurrence of kidney disease, nephrotoxicity of calcineurin inhibitors, and, most importantly, the presence or absence of rejection. Because T-cell-mediated rejection (TCMR) is characterized by inflammatory cell infiltration of the tubulointerstitium, we hypothesized that our findings would be useful to the early diagnosis of TCMR after kidney transplantation.

During this study, we measured uPSEP in patients who underwent a graft biopsy. Patients in the TCMR group were compared with those in the non-TCMR group to verify whether uPSEP is useful for diagnosing TCMR.

## Materials and methods

### Study design and settings

Of the patients who were receiving treatment at our facility and had undergone kidney transplantation, we targeted those who had undergone a graft biopsy in 2020 and 2023 and had preserved urine samples. We excluded protocol biopsy samples obtained at 1 h. Additionally, we targeted only the first biopsy if patients had undergone more than one.

This study was approved by the Institutional Review Board of Nagoya University Hospital (approval number: 2010–1135 and 2021–0379) and conducted in accordance with the Declaration of Helsinki. The kidney transplantations were all carried out in accordance with the Istanbul Declaration.

### Study samples and data collection

The attending physicians collected the demographic data, clinical history, medication history, and laboratory results of patients who had undergone a graft biopsy. The 24-h urine protein levels and/or urinary protein-to-creatinine ratios were measured. We also assessed for the presence of bacteriuria. If possible, blood and urine samples in addition to kidney tissue sections were obtained on the day of the kidney biopsy; then, samples were frozen and stored. We included cases for which urine samples could be collected on the day of the kidney biopsy.

### Presepsin assay

Frozen urine samples were thawed to a liquid state at room temperature before PSEP was measured. The uPSEP concentration was measured using a compact automated immunoanalyzer and a chemiluminescent enzyme immunoassay (PATHFAST, PHC IVD, Tokyo, Japan) [9, 10]. Measurements were performed according to the manufacturer’s instructions, and the results were obtained within approximately 17 min. The uPSEP levels were normalized to urinary creatinine levels and evaluated as uPSEP/creatinine levels (ng/gCr).

### Kidney tissue sections

Sections of kidney biopsy samples were prepared for light microscopy, immunofluorescence, and electron microscopy. Multiple (at least three) nephrologists reviewed the pathological specimens to establish the diagnosis. TCMR was diagnosed according to the 2022 Banff Classification for Renal Allograft Pathology [11]. As a general rule, CATEGORY 3 was not considered to be TCMR. However, in cases where there were individual circumstances, these were taken into account.

### Statistical analysis

Patient characteristics are presented as medians and interquartile ranges (IQRs) for continuous variables, and as numbers and percentages (%) for categorical variables. Patients were divided into the TCMR and non-TCMR groups and compared. Categorical variables were compared using Fisher’s exact test, and continuous variables were compared using the Mann–Whitney *U* test.

To detect TCMR, we conducted receiver-operating characteristic (ROC) curve analyses using uPSEP levels of patients who underwent biopsy. The area under the curve, sensitivity, and specificity were determined using the most discriminating thresholds.

To predict the presence of TCMR, we conducted a logistic regression analysis using uPSEP as an explanatory variable. The response variable was the presence of TCMR. The explanatory variable was adjusted for age and sex.

Data analyses were performed using R version 4.3.1 (R Foundation for Statistical Computing). All statistical tests were two sided, and *P* < 0.05 was considered statistically significant.

## Results

### Characteristics of study participants

A total of 39 patients (17 female and 22 male patients) with a median age of 57 years at the time of biopsy (IQR, 46.5–63 years) were included. The clinical characteristics of the patients are presented in Table 1. Only three of the 39 patients had TCMR. Of the three cases, two were classified as Grade I B. The remaining case involved a discrepancy in opinion as to whether the i score was 1 or 2, and the category was finally determined to be 3. However, in this case, TCMR was strongly suspected, and steroid pulse therapy was administered, and the kidney biopsy was performed one week after the steroid pulse therapy due to the New Year holidays. This case was determined to be TCMR based on the clinical situation. A significantly high proportion of patients with TCMR underwent an episode biopsy; however, of the eight patients who underwent an episode biopsy, more than half (five patients) did not have TCMR. Compared to the non-TCMR group, the TCMR group showed higher levels of serum creatinine. Four cases of bacteriuria were observed, but none of the patients were treated for urinary tract infection.

**Table 1:** Clinical characteristics in patients with kidney transplantation who were performed kidney biopsy

Factor	Without TCMR (*n* = 36)	With TCMR (*n* = 3)	*P* value
Sex (%)			
Female	16 (44.4)	1 (33.3)	1.000
Age at biopsy (y)	57.50 [50.25, 63.00]	43.00 [35.00, 51.50]	0.162
Age at transplant (y)	56.50 [46.50, 61.25]	43.00 [35.00, 47.00]	0.054
Duration from transplant (months)	9.50 [3.00, 12.00]	3.00 [1.50, 53.50]	0.648
Past history			
DM (%)	8 (22.2)	1 (33.3)	0.556
HT (%)	31 (86.1)	3 (100.0)	1.000
Episode biopsy (%)	5 (13.9)	3 (100.0)	0.006
ABO incompatible (%)	12 (34.3)	1 (33.3)	1.000
Cause of ESKD (%)			0.822
ADPKD	4 (11.1)	0 (0.0)	
AAV and GBM	1 (2.8)	0 (0.0)	
CGN	2 (5.6)	1 (33.3)	
cTIN	2 (5.6)	0 (0.0)	
DMN	5 (13.9)	0 (0.0)	
FSGS	4 (11.1)	1 (33.3)	
IgAN	5 (13.9)	0 (0.0)	
Malignant HT	1 (2.8)	0 (0.0)	
MN	1 (2.8)	0 (0.0)	
NSc	3 (8.3)	0 (0.0)	
Oligomeganephronia	1 (2.8)	0 (0.0)	
Unknown	7 (19.4)	1 (33.3)	
Deceased donor (%)	2 (5.6)	0 (0.0)	1.000
Dialysis (%)			0.190
Hemodialysis	13 (36.1)	1 (33.3)	
Peritoneal dialysis	1 (2.8)	1 (33.3)	
None, PEKT	22 (61.1)	1 (33.3)	
Body mass index (kg/m^2^)	21.78 [19.73, 24.23]	20.84 [19.14, 25.99]	0.958
Laboratory data			
Hb (g/dl)	11.85 [10.30, 12.67]	10.80 [10.10, 11.35]	0.268
ALB (g/dl)	4.00 [3.80, 4.20]	3.90 [3.85, 3.90]	0.474
Cr (mg/dl)	1.32 [1.10, 1.54]	2.93 [2.46, 3.37]	0.008
eGFR (ml/min/1.73m^2^)	40.15 [34.50, 46.92]	17.50 [15.60, 24.25]	0.011
CRP (mg/dl)	0.04 [0.02, 0.09]	0.16 [0.16, 0.16]	0.056
UPCR (g/day)	0.16 [0.10, 0.29]	0.31 [0.26, 0.88]	0.091
uPSEP (pg/mL)	505.00 [263.00, 1116.00]	5733.00 [4120.00, 11,196.00]	0.007
uPSEP (ng/gCr)	782.25 [336.44, 1278.93]	6788.63 [5374.57, 9931.87]	0.006
Banff Classification category (%)			< 0.001
Category 1	31 (86.1)	0 (0.0)	
Category 3	1 (2.8)	1 (33.3)	
Category 4	0 (0.0)	2 (66.7)	
Category 6	4 (11.1)	0 (0.0)	

TCMR, T-cell-mediated rejection; DM, diabetes mellitus; HT, hypertension; ESKD, end-stage kidney disease; ADPKD, autosomal dominant polycystic kidney disease; AAV, anti-neutrophil cytoplasmic antibody-associated vasculitis; GBM, anti-glomerular basement membrane disease; CGN, chronic glomerulonephritis; cTIN, chronic tubulointerstitial nephritis; DMN, diabetic nephropathy; FSGS, focal segmental glomerulosclerosis; IgAN, IgA nephropathy; MN, membranous nephropathy; NSc, nephrosclerosis; PEKT, pre-emptive kidney transplant; Hb, hemoglobin; ALB, albumin; Cr, creatinine; eGFR, estimated glomerular filtration rate; CRP, C-reactive protein; UPCR, urinary protein-to-creatinine ratio; uPSEP, urinary presepsin

### Comparison of uPSEP levels of patients with and without TCMR

**Fig. 1:**
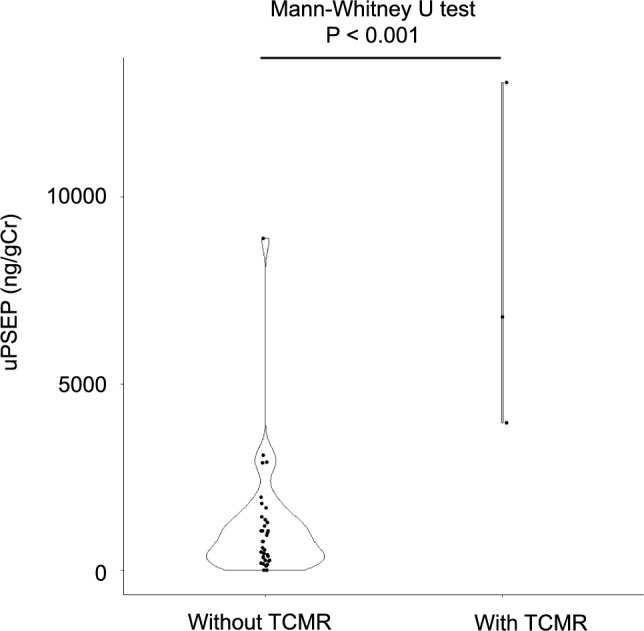
uPSEP levels of patients with and without TCMR. The uPSEP levels of patients with TCMR were significantly higher than those of patients without TCMR. TCMR, T-cell-mediated rejection; uPSEP, urinary presepsin

The uPSEP/creatinine levels of patients with TCMR were significantly higher than those of patients without TCMR (*P* < 0.001) (Fig. 1).

### Ability of uPSEP to detect TCMR

**Fig. 2:**
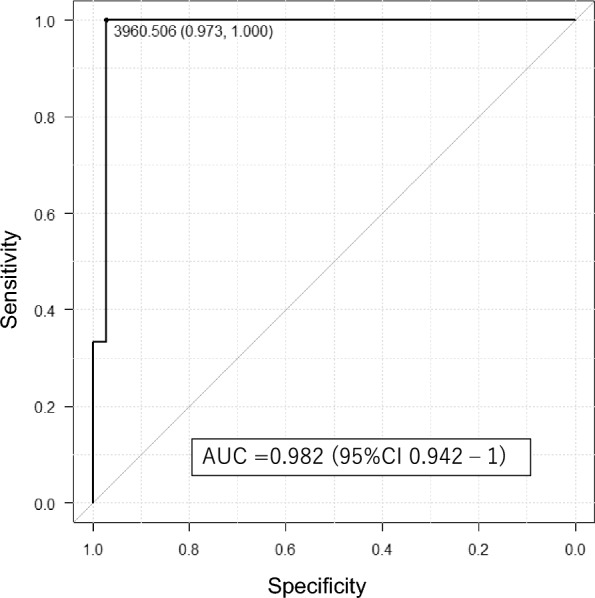
The ROC curve for the detection of TCMR in patients who underwent a kidney biopsy. The uPSEP level showed a high AUC, thus indicating its good ability to detect TCMR. AUC, area under the curve; ROC, receiver-operating characteristic; TCMR, T-cell-mediated rejection

We assessed the accuracy of TCMR detection using ROC curves. Among patients with TCMR, the area under the curve of uPSEP/creatinine was high (0.982), with a cutoff value of 3950 ng/gCr (Fig. 2).

### Odds ratios for the presence of TCMR based on uPSEP

To predict the presence of TCMR, we conducted a logistic regression analysis using uPSEP as an explanatory variable. Without adjustment, the odds ratio for TCMR per 1000-ng/gCr increase in uPSEP was 1.89 (95% CI, 1.12–3.21; *P* = 0.017). When adjusted for age and sex, the odds ratio was 1.90 (95% CI, 1.10–3.28; *P* = 0.22).

### Assessment of cases with elevated uPSEP but without TCMR

Case 1 involved a 60-year-old female patient who had experienced shingles and was administered amenamevir. Deterioration of the kidney function was not observed during hospitalization. She underwent a 3-month protocol biopsy a few days after antiviral drug treatment. The uPSEP value was 8892 ng/g Cr. The kidney sample was diagnosed as category 1 (normal biopsy or nonspecific changes) according to the 2022 Banff Classification for Renal Allograft Pathology.

Case 2 involved a 54-year-old female patient who experienced COVID-19 and was prescribed remdesivir. Deterioration in kidney function was not observed during hospitalization. She underwent a 1-year protocol biopsy a few days after antiviral drug treatment. The uPSEP value was 3093 ng/g Cr. The kidney sample was diagnosed as category 1 (normal biopsy or nonspecific changes) according to the 2022 Banff Classification for Renal Allograft Pathology.

## Discussion

This cross-sectional study aimed to determine whether uPSEP could be a useful predictor of TCMR, which is diagnosed according to kidney biopsy results. The results showed elevated uPSEP levels with TCMR.

To the best of our knowledge, this is the first study to show a link between uPSEP and TCMR. Previously, we reported a link between uPSEP and kidney disease [6]. However, PSEP was originally developed as a marker of infectious diseases in blood samples, and only one report of elevated uPSEP with pyelonephritis has been published [12]. Patients who have undergone kidney transplantation are at high risk for pyelonephritis; therefore, it is important to distinguish pyelonephritis from TCMR. This study does not include cases of symptomatic urinary tract infection or BK polyomavirus-associated nephropathy; therefore, their impact does not need to be considered.

In patients with other kidney diseases, uPSEP is produced by macrophages/monocytes in the kidney; therefore, we believe this same mechanism occurs with TCMR. We speculated that macrophages/monocytes produce PSEP in kidney tissue after tissue damage caused by TCMR occurs.

Patients can experience elevated uPSEP levels caused by conditions other than rejection; however, in these cases, serum creatinine levels were not significantly changed. Additionally, inflammatory cell infiltration was not clearly observed in the kidney biopsy samples. Therefore, a slight burden on the kidney tubules may occur before serum creatinine levels increase. Alternatively, it is possible that functional changes occurred before they were detected by pathological testing.

uPSEP measurements are noninvasive and can be performed repeatedly; therefore, monitoring this concentration enables an early diagnosis and the evaluation of treatment efficacy. Furthermore, because results can be obtained within a short time, they are clinically useful. Although the kidney biopsy sample reflects only a part of the entire kidney, it is possible that uPSEP reflects the entire kidney. Therefore, the ability of uPSEP to predict TCMR is promising.

This study had some limitations. First, the number of cases was limited because the frequency of TCMR is low. Second, uPSEP is thought to reflect inflammation of the tubulointerstitium in a nonspecific manner; therefore, if overlapping interstitial nephritis is present, then it may be difficult to distinguish the two. Third, uPSEP has been reported to be elevated in pyelonephritis; therefore, BK polyomavirus-associated nephropathy, one of the most critical complications related to kidney transplantation, may be challenging to distinguish from TCMR.

## Conclusion

The uPSEP level may predict TCMR with high accuracy in patients who have undergone kidney transplantation.

## Data Availability

All data analyzed during this research are included in this article. Reasonable requests for additional data or materials will be fulfilled under appropriate agreements. Requests should be made to the corresponding author.
